# Mental health and health behaviours among patients with eating disorders: a case–control study in France

**DOI:** 10.1186/s40337-022-00691-x

**Published:** 2022-11-10

**Authors:** Marie Galmiche, Clémence Godefroy, Najate Achamrah, Sébastien Grigioni, Guillaume Colange, Vanessa Folope, André Petit, Clément Rapp, Moise Coeffier, Pierre Dechelotte, Marie-Pierre Tavolacci

**Affiliations:** 1 UNIROUEN, Inserm U 1073, CHU Rouen, Department of Nutrition, Université de Rouen Normandie, 76000 Rouen, France; 2 CHU Rouen, Inserm CIC-CRB 1404, 76000 Rouen, France; 3 UNIROUEN, Inserm U 1073, CHU Rouen, Inserm CIC-CRB 1404, Université de Rouen Normandie, 76000 Rouen, France

## Abstract

**Background:**

Eating disorders (ED) are a public health concern due to their increasing prevalence and severe associated comorbidities. The aim of this study was to identify mental health and health behaviours associated with each form of EDs.

**Methods:**

A case–control study was performed: cases were patients with EDs managed for the first time in a specialized nutrition department and controls without EDs were matched on age and gender with cases. Participants of this study filled self-administered paper questionnaire (EDs group) or online questionnaire (non-ED group). Collected data explored socio-demographics, mental health including anxiety and depression, body image, life satisfaction, substances and internet use and presence of IBS (Irritable Bowel Syndrome).

**Results:**

248 ED patients (broad categories: 66 Restrictive, 22 Bulimic and 160 Compulsive) and 208 non-ED subjects were included in this study. Mean age was 36.0 (SD 13.0) and 34.8 (SD 11.6) in ED and non-ED groups, respectively. Among patients and non-ED subjects, 86.7% and 83.6% were female, respectively. Body Shape Questionnaire mean score was between 103.8 (SD 46.1) and 125.0 (SD 36.2) for EDs and non-ED group, respectively (*p* < 0.0001). ED patients had a higher risk of unsatisfactory friendly life, anxiety, depression and IBS than non-ED s (all *p* < 0.0001) Higher risk of anxiety, depression and IBS was found for the three categories of EDs. Higher risk of smoking was associated only with restrictive ED, while or assault history and alcohol abuse problems were associated only with bulimic ED. The risk of binge drinking was lower in all EDs categories than in non-ED.

**Conclusion:**

This study highlights the common comorbidities shared by all EDs patients and also identifies some specific features related to ED categories. These results should contribute to the conception of future screening and prevention programs in at risk young population as well as holistic care pathways for ED patients.

**Plain English summary:**

This case–control study evaluated mental health and health behaviours associated with the main categories of Eating Disorders (EDs). Cases were patients with EDs initiating care in a specialized nutrition department and controls without ED were matched on age and gender with cases. Self-administered paper questionnaires were filled by ED 248 patients (66 Restrictive, 22 Bulimic and 160 Compulsive) and online questionnaire by 241 non-ED controls. Body image satisfaction was significantly worse in ED patients than in controls. (*p* < 0.0001). Dissatisfactory life, anxiety, depression and irritable bowel syndrome were more found in patients with all EDs categories than in non-ED (*p* < 0.0001). Smoking risk was increased only in restrictive patients while and assault history and alcohol abuse was increased only in bulimic patients. These results highlight the global burden of ED and related comorbidities and provide useful information for future screening, prevention and care programs.

## Introduction

Eating disorders (EDs) are mental illnesses characterised by important disturbances of food behaviour, and body image [1], with significant physical and psychosocial associated impairments which result in increased morbidity and mortality. The Diagnostic and Statistical Manual of Mental Disorders (DSM-5) has described the different forms of specified EDs [2]: anorexia nervosa (AN), bulimia nervosa (BN) and binge eating disorder (BED) as referred as typical ED, while Other Specified Feeding or Eating Disorders (OSFED), including sub-syndromic or atypical forms of AN, BN and BED.A residual category of Unspecified Feeding or Eating Disorder has also been proposed. The major difference between BN and BED is the presence of compensatory behaviours in BN (purging behaviours including self-induced vomiting, laxative or diuretic abuse or fasting behaviours) but not BED. OSFED is a formal diagnostic category including restrictive, bulimic or compulsive behaviours that do not meet the full diagnostic criteria of typical AN, BN or BED, mainly a lower severity, frequency or duration. As an alternative to this strict DSM-5 distinction of typical ED and OSFED, a “broad categories” ED classification system has been proposed, based on the presence of restrictive, bulimic and compulsive behaviours [3, 4]. Based on the systematic review of 33 studies, the lifetime prevalence of EDs ranges from 3.3 to 18.6% for women and 0.8–6.5% for men, and has increased over recent years [5]. The lifetime prevalence of EDs differed according to sex and but also the type of ED, with the highest prevalence for BED (weighted mean of 2.8% for women and 1.0% for men), then BN (1.9% for women and 0.6% for min) and the lowest for AN (1.4% for women and 0.2% for men). Already with the DSM-IV classification, the prevalence of atypical ED was considered to be at least as high as that of typical ED [6]. Our recent review also reports that, using the DSM-5 classification, the prevalence of OSFED is higher than that of typical ED [5]. Using the broad categories also include other types of ED, the Unspecified Feeding and Eating disorders. Thus, the highest ED prevalence figures are that of broad categories. This high and increasing prevalence, combined with their important impact on quality of life, and risk of morbidity and mortality, makes EDs a major public health issue [7–9].

Some studies have been conducted on the different comorbidities associated with EDs, such as depression, anxiety or substance use [10–12]. Ulfvebrand et al. reported that 71% of patients with an ED showed one or more concurrent comorbid psychopathology (including mood disorders, anxiety disorders and substance-related disorders) and anxiety disorders were the most common comorbid psychopathologies for both male and female ED patients [13]. Some differences in comorbidities according to the form of ED have been reported, such as increased perfectionism in AN patients, whereas BED patients presented more frequently with addictive behaviours and psychological disorders [14]. Bahji et al. showed that substance use disorder was frequently reported comorbidity of EDs, with a prevalence ranging 13% to 27% according to the form of ED [15]. BN is the most prevalent ED associated to substance use disorders, followed by BED, AN and lastly OSFED [11, 15]. Internet addiction as a comorbid condition of EDs has been discussed more recently. One study did not find an association between EDs and the risk of internet addiction [16], while another one showed an association between body mass index (BMI) among women with ED and internet addiction [11]. Irritable bowel syndrome (IBS) presence is associated with body mass index in ED patients [17–19].

Many studies reported the characteristics of EDs, such as age of onset [20], risk factors [14], genetic factors [21] and personality [12, 22], either in all EDs or in separated groups of typical EDs. Nevertheless, few studies have compared psychiatric comorbidities and substance use of each EDs [11, 13, 15], and no study before included a comparison with age and sex-matched controls without EDs.

A better knowledge of comorbid conditions associated with any ED or some specific form of EDs is important to improve the early detection or the global care of the patient. Thus, to fill the above-mentioned gaps in the literature, this study was initiated with the hypothesis that risk or protective factors or comorbid conditions may differ depending onthe ED categories. The objectives of this study, performed in a large group of ED patients in comparison with non-ED controls, were: (1) to determine the prevalence of mental health conditions (life satisfaction, anxiety and depression) and health behaviours (alcohol abuse, smoking and cannabis use) according the category of ED; (2) to identify risk factors associated for each ED category.

## Methods

### Study design

This case–control study aimed to highlight the comorbidities associated with EDs. The ED patients were included in the Eating Disorders Inventory and Longitudinal Survey (EDILS) cohort [23]. Consecutive patients over 18 and under 70 years old, attending a first medical consultation in the Nutrition Department of a tertiary university hospital and, with an ED diagnosed by a clinician according to the DSM-5, were eligible for inclusion and invited to participate to this study. Patients agreeing to participate provided written informed consent and completed the first confidential, self-administered paper questionnaire. The patients included in this study were split into on the three broad categories of ED: restrictive, bulimic and compulsive. Restrictive EDs include typical and atypical anorexia nervosa, and restrictive ED other than typical or atypical AN, including ARFID and other residual restrictive USFED; the bulimic ED category includes typical and atypical bulimia nervosa, and purging behaviour; the compulsive ED category include typical and atypical binge eating disorders and night eating syndrome.

The control subjects were randomly recruited from the volunteer registry of the Clinical Investigation Center of the same university hospital. Controls filled out an anonymous, onlin (with LimeSurvey®) self-administered questionnaire, sent by mail between June and October 2021Volunteers aged 18 and 70 years were eligible for inclusion and screened for any EDs with the French version of the five-item “Sick, Control, One stone, Fat, Food” (SCOFF) questionnaire (Cronbach’s apha: 0.76) [24]. Two positive answers indicate a significant risk of ED; therefore, volunteers with two or more positive responses to SCOFF questionnaire were excluded. The control volunteers, with no detectable, were paired to patients according to the age class (18–25, 26–35, 36–50 and 51–70 years old) and the gender of the cases (ED patients). This age and sex-matched non-ED group serve as control group for comparisons.

### Data collection

The questionnaire recorded age, gender and living status (alone, with family/friends or as a couple). Body mass index was calculated using the weight and height measured on the day of inclusion. The weight and the height were objectively measured for the patients and self-reported by the control group. Major lifetime events such as death of a loved one, assault (sexual or physical) and abortion were collected.

### Mental health

Satisfaction with professional, friendship, marital and family life were assessed using five questions, with multiple-choice answers from “not at all satisfied” to “very satisfied”. Depression and anxiety were measured using the Hospital Anxiety and Depression Scale (HADS), which contains a set of seven questions each for anxiety and depression. A total score from 0 to 7 indicates a low risk of depression/anxiety, a total score from 8 to 10 indicates a probable diagnosis, and a total score higher than 11 indicates a certain diagnosis of depression/anxiety [24].

The BSQ-34 (Body Shape Questionnaire) (Cronbach’s alpha: 0.95) is 34-item self-report questionnaire evaluating body image and body dissatisfaction [25]. The BSQ identifies an excessive preoccupation about bodily appearance, exploring its involvement in the onset and/or perpetuation of EDs. According to the cut-off points proposed by Cordás and Castilho, higher scores reflect higher bodily concerns, and the scale is scored from 34 to 204 [26]. A score below 80 indicates no dissatisfaction, between 80 and 110 slight dissatisfaction, between 111 and 140 moderate dissatisfaction, and higher than 140 serious dissatisfaction [27].

### Health behaviours

Tobacco status was registered as follows: non-smoker, former smoker and smoker (at least one cigarette per day). The Fagerström test was used to assess the tobacco dependence level [28] and data were classified into three groups: weak, medium and strong. Cannabis use was defined as occasional, with at least one episode of consumption in the previous 12 months, or regular, with at least 10 episodes in the last month; occasional and regular cannabis use were both categorised as cannabis use.

Alcohol use disorders were assessed using the Alcohol Use Disorders Identification Test (AUDIT) (Cronbach’s alpha: 0.83) [29]. Alcohol disorders were recorded as follow: no disorders, misuse, and risk of disorders; misuse and risk of disorders were categorised as alcohol abuse problems (a score of 7 or more for women and 8 or more for men). Binge drinking was defined as the consumption of five or more alcoholic drinks in less than 2 h; occasional episodes was defined by once or less per month and frequent as twice or more per month.

The Orman test was used to assess internet dependence risk (Cronbach’s alpha 0.92) Total score was used to classify participants into three groups: 0–3 as low dependence risk; 4–6 as moderate dependence risk; and 7–9 as high dependence risk.

Irritable bowel syndrome (IBS) was assessed using binary questions of the ROME III criteria. These criteria include recurrent abdominal pain or discomfort (at least 3 days per month in the previous 3 months) associated with two or more of the following: improved by defecation; onset associated with a change in stool frequency, and; onset associated with a change in form (appearance) of stool.

### Statistical analysis

The chi-square (χ^2^) test was used for comparisons of discrete data. Continuous variables were summarised using means and compared using Student’s *t*-test. All factors with a *p*-value lower than 0.20 in the *t*-tests were integrated into the multivariate logistic regression model. Multivariate logistic regression was performed to identify mental health and health behaviour factors associated with EDs. Adjusted odds ratios (AOR) and their 95% confidence intervals (CI) were calculated. Bonferroni's post-hoc correction was performed for univariate analysis so with a *p* < 0.01 as significant. The non-ED control served as the reference group for analysis. Categories of answers were grouped:—Probable and certainty anxiety and depression were respectively classified as anxiety and depression; Moderately unsatisfied and unsatisfied professional, friendship, marital and family life categorized as unsatisfied; Frequent and occasional binge drinking as binge drinking;—misuse and risk use of alcohol as alcohol abuse problem; medium and strong answers to the Fagerström test as nicotine dependence;—moderate and high answers from the Orman test as cyberaddiction. The anxiety and depression variables could not be calculated for bulimic EDs because the sample size did not respect the validity conditions of the logistic regression. Analyses were performed using Xlstat (2020.3.1).

## Results

**Fig. 1:**
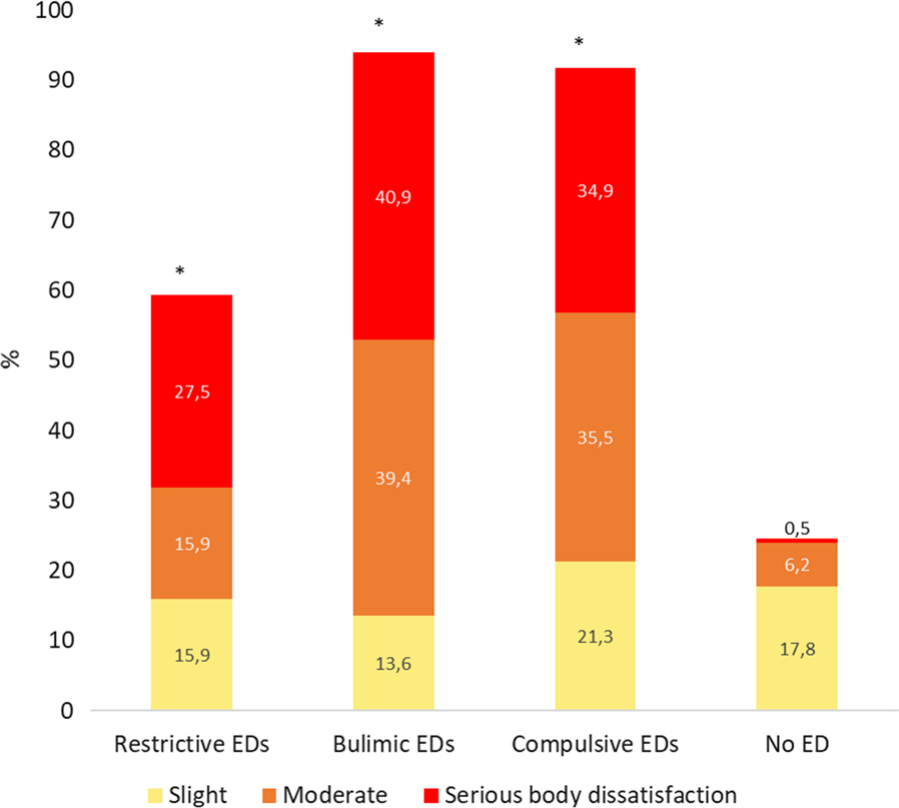
Body dissatisfaction according the eating disorders group (n = 242) or no eating disorders (n = 208). *p < 0.01 each ED category compared to non-ED group

**Fig. 2:**
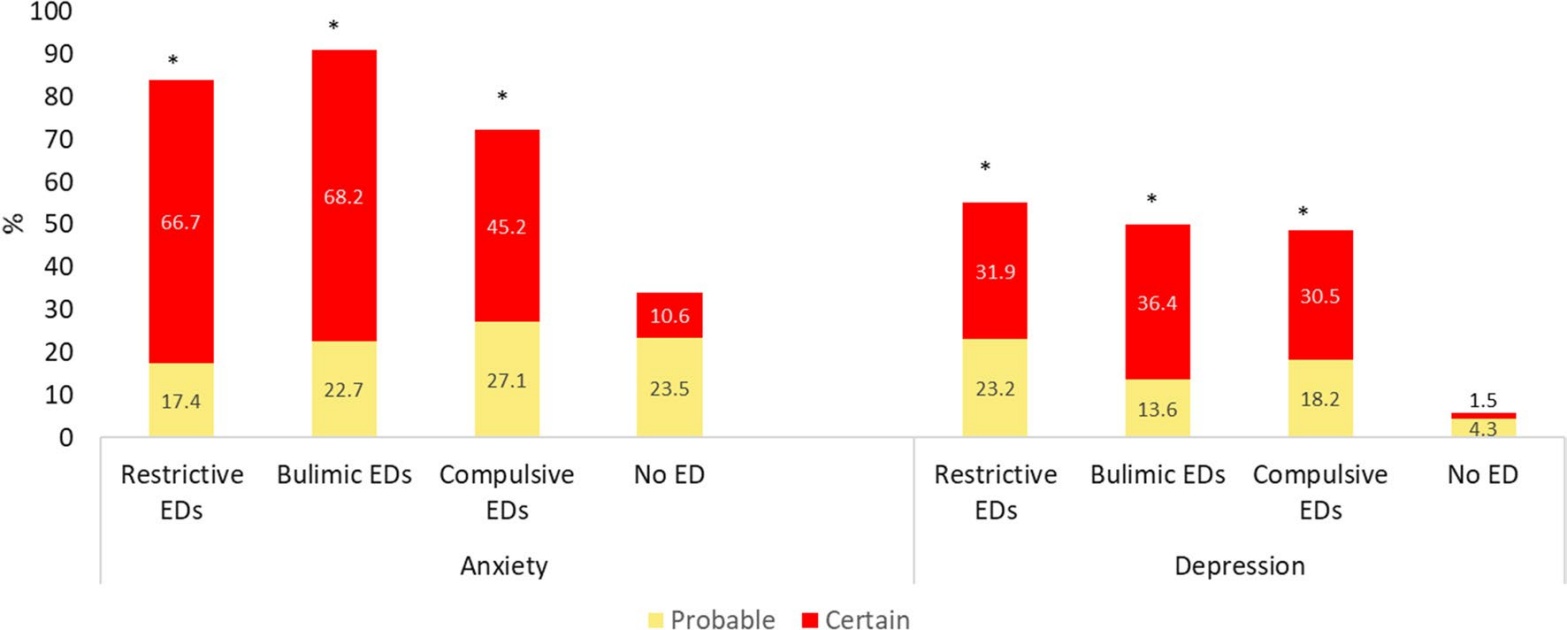
Anxiety and depression according to the group of eating disorders (n = 242) or no eating disorders (n = 208). *p < 0.01 each ED category compared to non- ED group

**Fig. 3:**
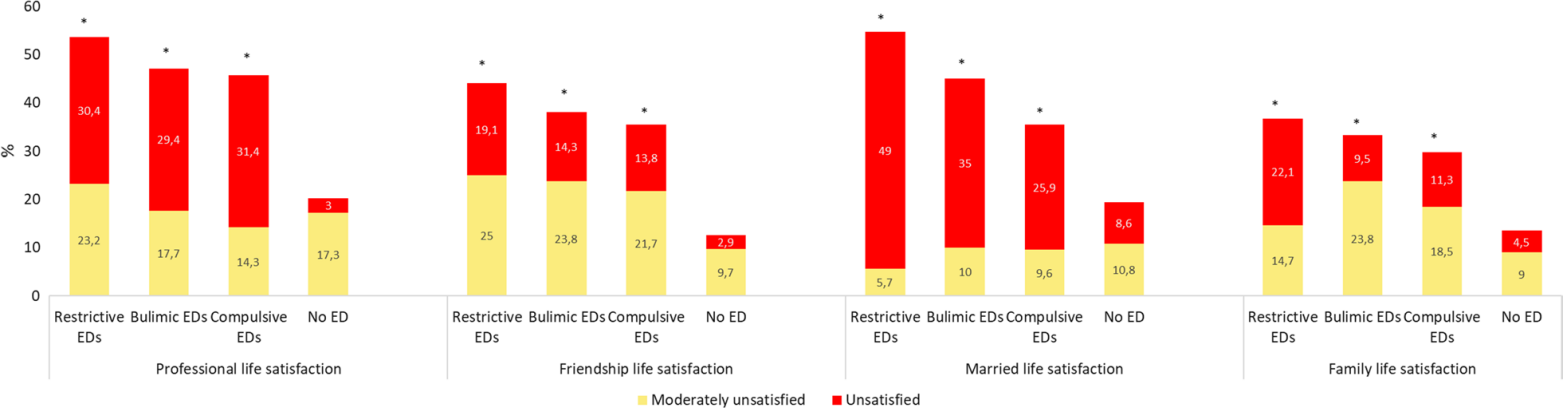
Life satisfaction according to the group of eating disorders (n = 242) or no eating disorders (n = 208). *p < 0.01 each ED category compared to non- ED group

**Fig. 4:**
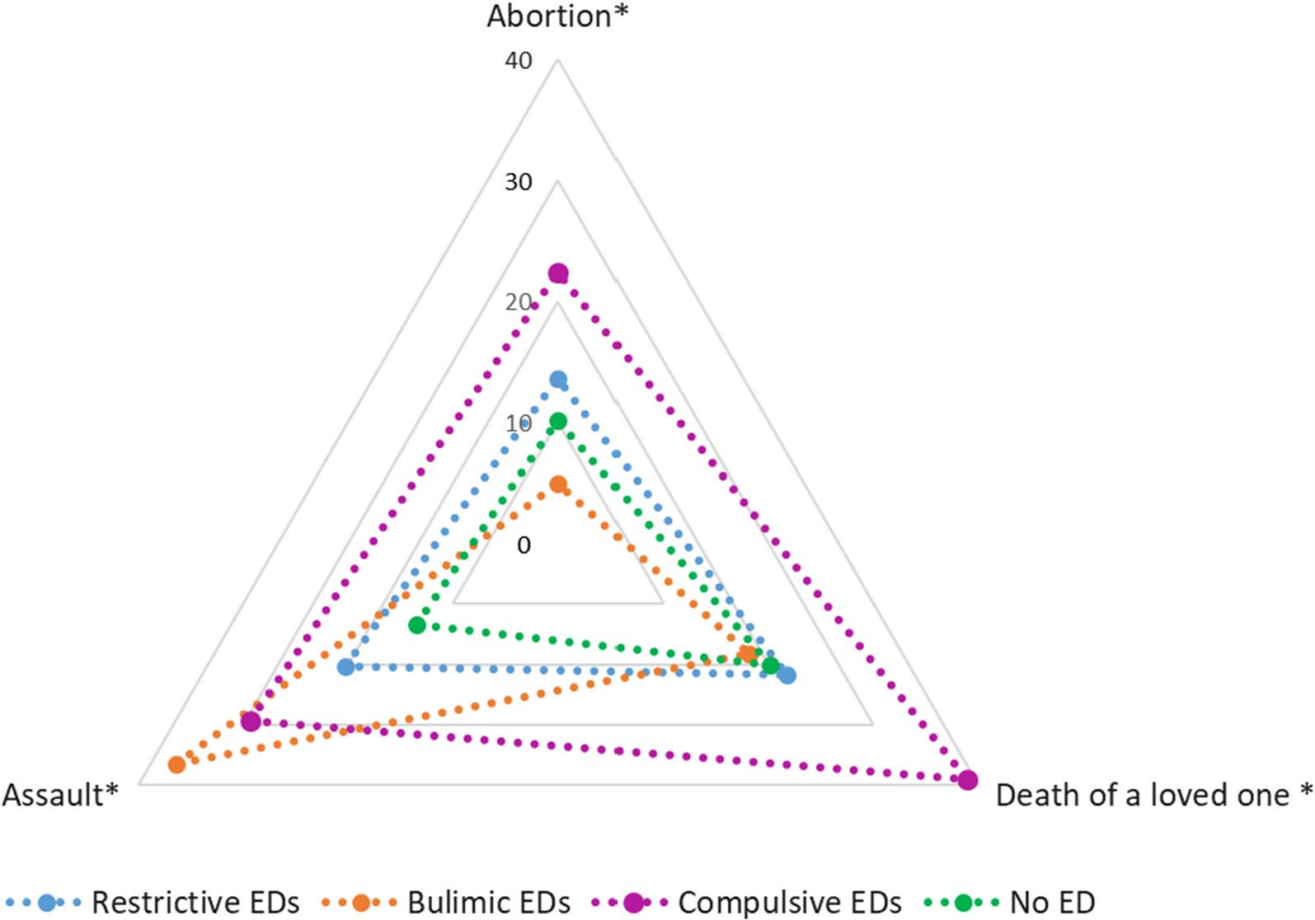
Major life event according to the group of eating disorders (n = 242) or no eating disorders (n = 208). *p < 0.01 each ED category compared to non- ED group

**Fig. 5:**
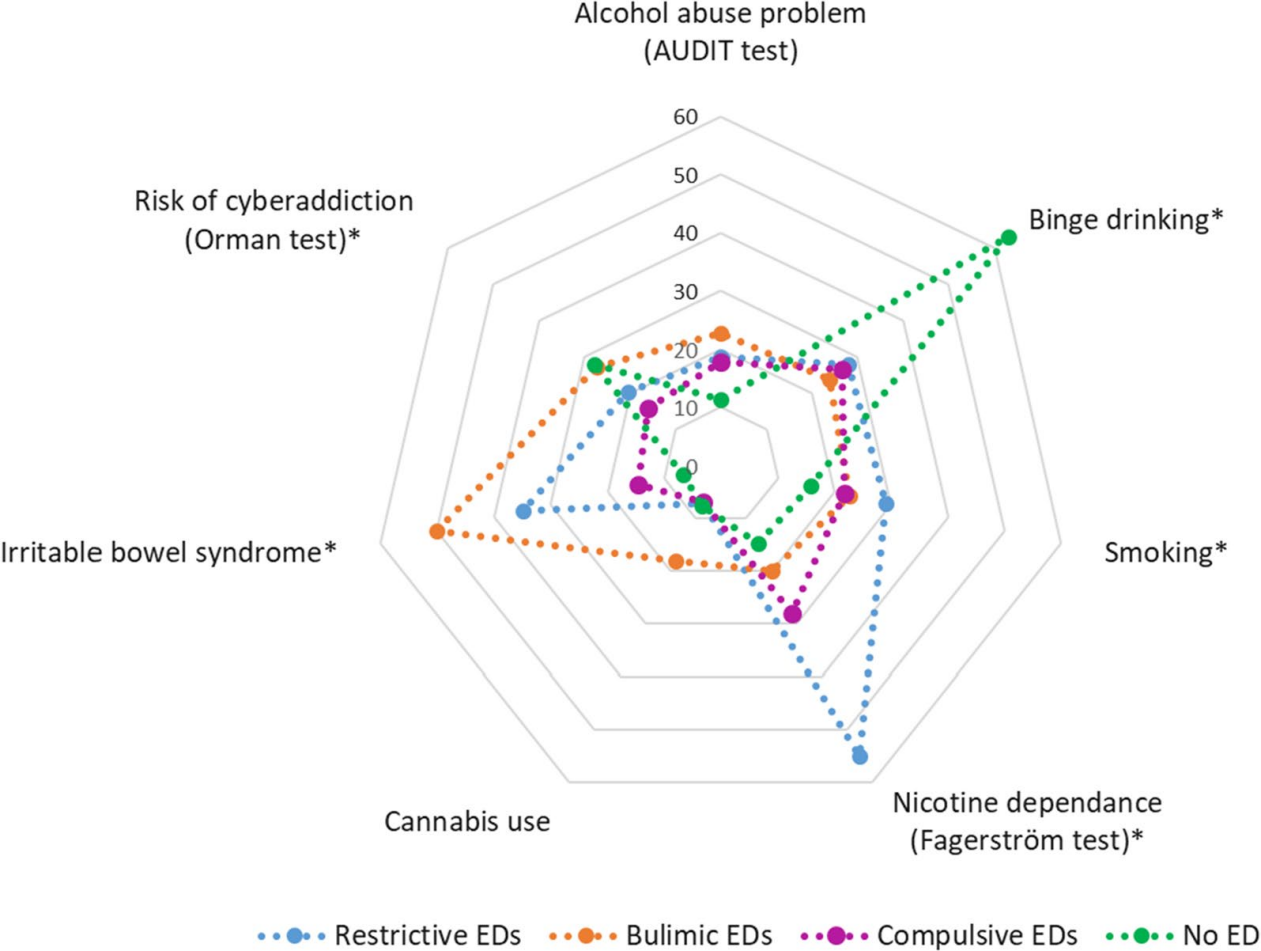
Health behaviors according to the group of eating disorders (n = 242) or no eating disorders (n = 208). *p < 0.05 each ED category compared to non- ED group

In total, 248 patients with EDs were included (participation rate almost 30%), with 22 having bulimic, 66 restrictive and 160 compulsive disorders. The online self-questionnaire was filled out by 241 control group (response rate: 58%) , of which 33 (13.7%) were excluded due to a positive SCOFF. Finally, 208 non-ED were included in the control group. Age, gender and BMI are described in Table 1. The mean BSQ score was 103.8 (Standard Deviation (*SD*) 46.1) among the patients with restrictive EDs, 125.0 (*SD* 36.2) for bulimic EDs, 123.8 (SD 34.7) for compulsive EDs and 66.8 (*SD* 25.3) for non-ED (*p* < 0.0001). Serious body dissatisfaction was higher among the patients with EDs than in the non-ED group (*p* < 0.0001) (Fig. 1). Anxiety was present at a prevalence of 45.2–66.7% depending on the category of ED, and depression from 30.5 to 36.4% (Fig. 2). These disorders were positively correlated with BSQ (r = 0.53, *p* < 0.0001; and r = 0.52, *p* < 0.0001; respectively). Life dissatisfaction scores are presented in Fig. 3. For all types of life dissatisfaction (professional, friendship, marital and family), dissatisfaction scores were higher among patients with EDs than in the non-ED group (*p* < 0.0001). The highest dissatisfaction scores were related to marital life. Concerning major life events, abortion was reported at a prevalence from 5.0 to 22.4% according to the category of ED, death of a loved one from 18.2 to 38.9%, and assault from 20.3 to 29.3% (Fig. 4). Figure 5 shows the prevalence of each health behaviour and risk, and major health concerns. The prevalence of irritable bowel syndrome was up to 50.0%.

**Table 1:** Description of the participants according to the group of eating disorders

	Restrictive EDs (*n* = 66)	Bulimic EDs (*n* = 22)	Compulsive EDs (*n* = 160)	*p*	EDs (*n* = 248)	No EDs (*n* = 208)	*p*
*Sex*							
Female (%)	95.7	95.5	81.5	0.007	86.7	83.7	0.36
Age mean (SD)	31.5 (12.2)	32.0 (13.6)	38.5 (12.6)	0.0001	36.0 (13.0)	34.8 (11.6)	0.30
Median (Q-Q3)	26 (21–40)	26 (24–35)	38 (28–47)		31 (27–39)		
*BMI*							
< 18.5 (%)	87.9	9.1	0.0	< 0.0001	24.2	1.9	< 0.0001
18.5–24.9 (%)	12.1	63.7	2.5		10.1	72.1	
25–29.9 (%)	0.0	13.6	10.0		8.0	19.7	
≥ 30 (%)	0.0	13.6	87.5		57.7	6.3	
BSQ mean (SD)	101.8 (44.8)	125.0 (36.3)	126.2 (36.1))	0.001	119.4 (37.8)	66.8 (25.3)	< 0.0001

According to the logistic regression analysis (Table 2), anxiety and depression were positively associated with all forms of ED. Dissatisfaction with professional life was positively associated with restrictive and compulsive EDs, and dissatisfaction with friendship with all forms of ED. Abortion and death of a loved one were associated with a higher risk of compulsive EDs, and assault with bulimic and compulsive EDs. Smoking was more frequent among those with restrictive EDs compared to the control population, and IBS was more frequent in all categories of ED. Binge drinking was negatively associated with all categories of ED, and the risk of cyberaddiction was lower for the compulsive EDs.

**Table 2:** Associated factors according to the group of eating disorders: univariate and multivariate analysis (reference group: no eating disorders)

	Restrictive EDs AOR (CI95%)	Bulimic EDs AOR (CI95%)	Compulsive EDs AOR (CI95%)
Anxiety	**5.57 (2.38–13.03)**	–	**4.08 (2.35–7.09)**
Depression	**13.14 (4.95–34.88)**	–	**8.16 (4.04–16.48)**
*Unsatisfied life*			
Professional	**3.61 (1.56–8.38)**	2.73 (0.78–9.61)	**2.16 (1.22–3.84)**
Friendly	**1.17 (1.60–10.55)**	**5.43 (1.32–22.36)**	**2.30 (1.10–4.80)**
Marital	2.48 (0.93–6.61)	3.55 (0.85–14.75)	0.97 (0.48–1.96)
Family	1.51 (0.54–4.19)	0.96 (0.20–4.68)	1.66 (0.77–3.58)
*Life event*			
Abortion*	1.31 (0.49—3.50))	0.54 (0.07–4.3)	**2.27 (1.18–4.37)**
Death of love one	1.52 (0.67–3.44)	1.19 (0.33–4.19)	**2.29 (1.34–3.91)**
Assault	1.73 (0.79–3.75)	**3.75 (1.41–9.97)**	**2.98 (1.66–5.06)**
*Health behavior*			
Binge drinking	**0.25 (0.12–0.49)**	**0.19 (0.07–0.54)**	**0.20 (0.12–0.32)**
Alcohol abuse problem	1.59 (0.69–3.68)	2.14 (0.67–6.85)	**1.92 (1.02–3.64)**
Smoker	**2.17 (1.04–4.54**)	1.44 (0.46–4.55)	1.76 (0.98–3.16)
Cannabis use	0.55 (0.15–2.10)	1.88 (0.46–7.61)	1.05 (0.44–2.49)
Cyberaddiction risk	0.63 (0.30–1.35)	0.97 (0.34–1.76)	**0.52 (0.30–0.92)**
Irritable bowel syndrome	**7.88 (3.41–18.23)**	**13.38 (4.56–39.38)**	**2.84 (1.35–6.01)**

Bold values indicate significant results* p* < 0.05

*Among women

## Discussion

To our knowledge, this study is the first to report mental health and health behaviours among ED patients compared to non-EDs group (non-ED). First, this study highlights the factors and comorbidities associated with three broad categories of EDs. In accordance with the literature, our study population was predominantly female [5, 30]. On average, the compulsive EDs group was older than the two other ED groups. This difference can be explained in part by the duration between the estimated beginning of the pathology and the first consultation, which is significantly longer in the compulsive ED group (10 years) compared to restrictive (5 years) and bulimic (7 years) ED groups. The difference in age of onset (younger in AN and BN patients) may be another explanation of the higher average age in the compulsive EDs group [19, 31].

Our study shows that depression and anxiety are more frequent in the ED group, for all types of ED, than in the non-ED group, which is in accordance with the literature [32, 33]. Elran-Barak et al. showed that the presence of anxiety symptoms, depression symptoms or both had an impact on ED symptoms, demonstrating the importance of psychiatric comorbidity in EDs [32].

Social relationships among patients with EDs are poorly documented in recent literature. Our evaluation of life satisfaction according to four items (professional, friendship, marital and family) provided an overview of this data and allowed us to identify the main fragile social interactions for each ED type as reported among women with BN [34]. Across all types of ED, patients were more dissatisfied about their friendship life than the non-ED group. The restrictive and compulsive groups were significantly more dissatisfied about their professional life than the non-ED group.

In our study, assault and death of a loved one were factors associated with compulsive EDs. Assault was a factor associated with bulimic EDs, but there was no life event significantly associated with restrictive EDs. Lie et al. [35] showed in their study that the anorexia nervosa restricting subtype (group did not differ significantly from the control group in overall stressful life events). Bereavement was significantly more common in the compulsive as we reported but also in the bulimic group). Finally, both non-sexual and sexual event were associated with binge eating and purging EDs. Hilbert et al. also showed in their study that sexual and physical abuse are risk factors of eating disorders, especially for BED and BN [14].

The association between EDs and other addictive behaviours has been established in the literature, with a stronger association in patients with BN than with AN [36]. Addictions may present a particular risk for BN patients. This was confirmed in another study, which highlighted the link between EDs and cannabis use [37]; we observed a higher prevalence of occasional cannabis use in the bulimic ED group compared to the others. Smoking is a factor associated with the restrictive ED group in accordance with the literature [11].

Concerning alcohol use, our results for the ED group did not differ significantly between the restrictive, bulimic and compulsive ED groups. However, we did observe a much higher binge drinking prevalence in the non-EDs group (63.2%) as compared to ED patients, which was between 23.8 and 27.9%. This phenomenon could be explained by the social context favouring binge drinking in young adults; indeed, binge drinking is often associated with spending time on festive occasions with friends, who drink frequently and do not comply with the drinking norms observed in the wider social environment [38]; in contrast the ED group had fewer social relationships or social relationships of lower quality than non-EDs group, as shown above. Chronic reward signalling elicited by excessive food intake in compulsive patients may also contribute to install food addiction and limit the use of binge drinking to reinforce dopamine release [39]. This may explain why binge drinking was associated with a decreased risk of EDs in our study. The reduced prevalence of binge drinking in ED patients does not mean the absence of any alcohol-related problems in these patients. Indeed, regarding AUDIT results, the compulsive group was at a significantly higher risk of alcohol use problems compared to control. The increases seen in the other ED groups were not significant, probably due to the small sample sizes in these groups, but the results follow the same trend. It should be underlined that AUDIT and binge drinking detect different behaviors in relation to alcohol use. Udo et al. showed in their study that alcohol use disorder is a risk factor associated with all ED groups [40]. Finally, there might be some issue of temporal change in behaviour that may not be detected by the questionnaires, patients switching from an addiction to another over time, excess alcohol intake preceding or following phases of acute disordered eating.

Concerning the association of cyberaddiction with EDs, our study showed a negative association (significant for compulsive and following the trend for restrictive and bulimic). Cyberaddiction had a higher prevalence in the non-ED group (27.9%) than the ED groups (from 15.9% for compulsive to 27.3% for bulimic). Our results were different than those in the literature [41]. A large number of studies on this topic showed the link between cyberaddiction and EDs using questionnaires or validated tests for screening EDs. Thus, the population studied were not necessarily ED patients with a medical diagnosis and medical follow-up [42]. The results of our study could indicate that cyberaddiction has a real impact in the onset of EDs, but, once diagnosis of ED, ED patients reduce their internet use. For further studies concerning cyberaddiction or problematic internet use and EDs, we suggest that attention is paid to the timing of ED onset in relation to cyberaddiction.

IBS is also tightly associated with all ED groups, especially for bulimic ED patients in our study. Dejong et al. also found an high prevalence of IBS in their bulimic patients (68.8%); however, they used the Manning criteria as the measurement instrument [43]. Conversely, Udo et al. showed that BED and AN were highly associated with bowel problems, but BN was not. Moreover, only 8.4% of the BN group presented with a bowel problem (including inflammatory bowel disease and IBS), compared to 8.0% for the AN group, 11.9% for the BED group and 3.7% for the control group [40]. About 23% of our ED study population presented a Rome III diagnosis of IBS, which is more than three times the 6.7% prevalence of our non-EDs study population, and twice the 11% prevalence calculated in a similar (age- and sex-ratio-matched) population from data published on the general population [44]. Other studies have already identified the high prevalence of IBS in patients with EDs; vice-versa, patients presenting to their GP with digestive symptoms are more likely to have EDs [45, 46]. Perkins et al. show that 87.6% of the participants in their study had an onset of their EDs before IBS symptoms, but for 6.7% it was the contrary and 5.6% of the participants began experiencing IBS and the ED at the same time [18].

### Strengths and limitations

One of the strengths of our study is the size of the sample of patients (n = 248), allowing comparison of the three broad categories of ED with a group of non-EDs group. Despite this, there is a limitation concerning the low numbers of patients with bulimic EDs, with a lake of power in the multivariate analysis. Another limitation is the self-reported nature of the data. The convenience sample of the patients could be a selection bias however the percentage of women and the mean age is not different of the total patients population. Control group were randomly recruited from the volunteer registry of the Clinical Investigation Center in the same area of the patient The response rate of 58% could be a selection bias. However, studies have shown that the self-report method may be preferable in some ways; for example, the detection of complex features such as binge eating and body image concerns is usually better with the self-report method [47].

## Conclusion

This study demonstrates that many features and specific comorbidities are shared across the spectrum of ED (anxiety, depression, friendship life dissatisfaction and IBS), and others were more specific to a particular ED group, such as smoking or major life events. These results highlight the support needs of these patients in addition to those regarding their ED, with particularities depending on the specific ED. Altogether, we think that results of this study enable to get a more comprehensive and global approach of the patients with ED that may be useful for future screening, prevention and care programs. Especially in patients with non-typical ED, the reduced severity or frequency of pathological behaviours may reduce the ability to detect the ED in the primary care setting. In this case, the combination of several clinical indicators related to comorbid conditions and risk factors may help to detect the ED. Similarly, in patients with no established ED but with identified risk factors or comorbid conditions, interventions programs could be proposed to reduce the risk of developing a subsequent ED. Finally, when ED is already patent, attention should be dedicated to a holistic care program addressing all comorbid conditions to interrupt the vicious circle of reinforcement between the ED and the comorbid conditions.

In order to better understand the care pathway of patients with ED, timing of the risk factors and of the ED, the mortality outcome, it would be worthwhile to conduct a cohort study using the French administrative health care database (SNDS) link with the self-questionnaire.

## Data Availability

On request.
